# Socioeconomic variations in access to smoking cessation interventions in UK primary care: insights using the Mosaic classification in a large dataset of primary care records

**DOI:** 10.1186/1471-2458-13-546

**Published:** 2013-06-05

**Authors:** Laura Douglas, Lisa Szatkowski

**Affiliations:** 1 UK Centre for Tobacco Control Studies, University of Nottingham, Division of Epidemiology and Public Health, Clinical Sciences Building, Nottingham City Hospital, Nottingham NG5 1PB, UK

## Abstract

**Background:**

Smoking prevalence is particularly high amongst more deprived social groups. This cross-sectional study uses the Mosaic classification to explore socioeconomic variations in the delivery and/or uptake of cessation interventions in UK primary care.

**Methods:**

Data from 460,938 smokers registered in The Health Improvement Network between 2008 and 2010 were analysed. Logistic regression was used to calculate odds ratios for smokers having a record of receiving cessation advice or a prescription for a cessation medication during the study period by Townsend quintile and for each of the 11 Mosaic groups and 61 Mosaic types. Both of these measures are area-level indicators of deprivation. Profiles of Mosaic categories were used to suggest ways to target specific groups to increase the provision of cessation support.

**Results:**

Odds ratios for smokers having a record of advice or a prescription increased with increasing Townsend deprivation quintile. Similarly, smokers in more deprived Mosaic groups and types were more likely to have a documented cessation intervention. The odds of smokers receiving cessation advice if they have uncertain employment and live in social housing in deprived areas were 35% higher than the odds for successful professionals living in desirable areas (odds ratio (OR) 1.35, 95% confidence interval (CI) 1.20-1.52; absolute risks 57.2% and 50.1% respectively), and those in low-income families living in estate-based social housing were 50% more likely to receive a prescription than these successful professionals (OR 1.50, 95% CI 1.31-1.73; absolute risks 19.5% and 13% respectively). Smokers who did not receive interventions were generally well educated, financially successful, married with no children, read broadsheet newspapers and had broadband internet access.

**Conclusions:**

Wide socioeconomic variations exist in the delivery and/or uptake of smoking cessation interventions in UK primary care, though encouragingly the direction of this variation may help to reduce smoking prevalence-related socioeconomic inequalities in health. Groups with particularly low intervention rates may be best targeted through broadsheet media, the internet and perhaps workplace-based interventions in order to increase the delivery and uptake of effective quit support.

## Background

Smoking is the leading cause of preventable illness in the UK [1], removing, on average, ten years from a smoker’s life [2]. Whilst the population prevalence of smoking has decreased over time, prevalence remains high in more socially deprived groups [3,4]. In the UK in 2010, smoking prevalence was 28% in adults living in households where the main earner worked in a manual or routine occupation, compared to 13% where the main earner was in a managerial or professional occupation [5]. A recent study using the Mosaic geo-demographic classification [6] (a measure of social group incorporating a wide range of individual and area-level characteristics) demonstrated even wider disparities in smoking prevalence; in some sectors of the population as many as 43% of adults smoked and in others the figure was as low as 9% [7].

Approximately two-thirds of smokers want to quit smoking, a figure similar across all social groups [5]. However, the most deprived smokers are three times less likely to succeed in quitting than the least deprived (OR 2.5; 95% CI 1.4-4.7) [8]. Most previous studies have primarily defined participants’ social group using occupational measures or area-level composite indicators such as the Townsend score [9], which combines census data on unemployment, car ownership, housing tenure and overcrowding for output areas of approximately 150 houses. However, studies have highlighted a need to better understand a range of other factors associated with quitting [10,11], such as living in a smoke-free household [12], the area that the person lives [10], and support available from family and friends [13]. A more nuanced understanding of the factors associated with smoking and quitting behaviour may allow smoking intervention programmes to better target individuals and increase quit and quit success rates.

General practitioners (GPs) are well-placed to encourage and support smokers to quit by offering interventions which have been proven effective and cost-effective, such as delivering brief cessation advice [14] or offering a prescription for nicotine replacement therapy (NRT) [15], bupropion [16] or varenicline [17]. This study builds on previous work [7] and uses Townsend quintile alongside the Mosaic classification to explore which particular groups are most and least likely to be offered and/or receive cessation support from a GP and to suggest ways in which particular groups can be targeted to increase the use of these effective cessation interventions.

## Methods

### Data source

This study utilises data from The Health Improvement Network (THIN), a dataset of medical records from approximately six million patients in 479 general practices throughout the UK [18]. THIN has previously been shown to be demographically representative of the UK population [19], and recorded prescribing rates of smoking cessation medications are similar to dispensing rates of these medications in pharmacies [20]. This study uses data from 460,938 patients aged 16+ who were registered in THIN for a two year period from 1st July 2008 to 31st June 2010. All patients had one or more Read codes [21] indicating they were a current smoker recorded in their medical records during the study period, and were therefore potentially eligible to receive a smoking cessation intervention.

Read codes were used to identify patients with one or more records of cessation advice in their medical notes in the two year study period, and British National Formulary (BNF) [22] codes were used to ascertain patients with one or more prescriptions for NRT, bupropion or varenicline. The absence of a record does not necessarily indicate that the intervention was not offered by the GP, but perhaps only that it was offered by the clinician but declined by the patient. Other data extracted were patients’ age, sex, Townsend quintile, Mosaic classification and the presence of smoking-related chronic disease (asthma, coronary heart disease, chronic obstructive pulmonary disease, diabetes mellitus, hypertension, stroke or transient ischaemic attack, psychoses and chronic kidney disease). Patients were classified as having one of these chronic conditions if they had a relevant Read code in their medical notes before the start of, or during, the study period (plus, in the case of asthma, a relevant prescription indication treatment of active asthma during, or within one year before, the start of the study).

### Mosaic

Mosaic is a geo-demographic profiling system, developed by Experian to help businesses understand their customers [6]. Multivariate modelling is used to group all of the UK’s 1.4 million postcodes into one of 61 Mosaic types and 11 aggregated broader groups, at the level of the full postcode (equating to 15 households on average across the country). Modelling utilises over 400 variables from a range of sources – 54% of data are derived from the national Census, whilst the remaining 46% include data from a range of sources, such as the Mosaic UK Consumer Dynamics Database, Experian Lifestyle Survey, Post Office Address File, shopping centre data and Land Registry data [6]. Due to commercial sensitivities some data sources are not disclosed, and details of the modelling procedure remain classified. Mosaic data were provided by Experian and anonymously linked to THIN by EPIC, who provided the THIN data.

### Analyses

The proportion of smokers who received cessation advice or a prescription for a smoking cessation medication was calculated for each quintile of Townsend score, and each of the 11 Mosaic groups and 61 Mosaic types. Logistic regression was used to calculate the odds of smokers receiving these interventions, both unadjusted and adjusted for age, sex and the presence of smoking-related chronic conditions, and accounting for the clustering of patients within practices using a cluster sandwich estimator. In the case of medication prescribing, odds ratios were additionally adjusted for whether the patient had also received cessation advice, as arguably if the smoker has received advice to quit they may be more likely to be prescribed a smoking cessation aid. We used likelihood ratio tests with a p value of < 0.05 to select confounders for inclusion in the multivariable models. Ethical approval for the use of the THIN data was granted by the EPIC Scientific Review Committee, and all analyses were completed using STATA version 11.0 (STATA Corp, College Station, TX).

The Mosaic Multimedia Guide [23] was used to describe and compare the characteristics of the Mosaic types found to be most and least likely to receive smoking cessation advice and prescriptions for cessation medications, including characteristics such as education, benefits received, occupation, household income and exposure to communications media such as newspapers, internet access and time spent watching television.

## Results

Of the 460,938 smokers included in this study, 51.3% were male, their average age was 45 (range 16–102, interquartile range 31–58) and 37.0% had one or more smoking-related chronic conditions. A Townsend quintile was available for 96.4% of smokers, and Mosaic classification for 80.6%. Overall, 53.1% of smokers received advice to quit during the two year study period, and 16.5% of smokers received one or more prescriptions for a smoking cessation medication.

Table 1 shows the proportion of smokers in each Townsend quintile who received advice to quit or a smoking cessation medication during the study period, with odds ratios presented with reference to the least deprived group. Adjustment for confounders had no appreciable effect on the magnitude and direction of the unadjusted odds ratios, and so for brevity only adjusted ratios are presented.

**Table 1:** Odds ratios for the association between Townsend quintile and receiving smoking cessation interventions (UK, 2008–10)

**Townsend Quintile**	**n**	**Advice**			**Prescription**		
		**% received**	OR* (95% CI)	**P-value for trend**	**% received**	OR** (95% CI)	**P-value for trend**
1 (Least deprived)	78,338	50.3	1.00	-	14.7	1.00	-
2	81,865	51.6	1.05 (1.01-1.08)	< 0.001	15.5	1.05 (1.00-1.10)	0.001
3	95,451	52.6	1.10 (1.05-1.15)		16.7	1.13 (1.05-1.21)	
4	101,648	54.4	1.19 (1.12-1.26)		17.6	1.18 (1.10-1.27)	
5 (Most deprived)	87,138	56.4	1.28 (1.19-1.37)		17.5	1.16 (1.05-1.28)	
Missing	16,498	50.9	1.08 (0.98-1.18)	0.132	16.7	1.12 (0.97-1.30)	0.111

*adjusted for age, gender and chronic condition **adjusted for age, gender, chronic condition and advice given.

*OR* = odds ratio; 95% CI = 95% confidence interval.

Table 1 shows a statistically significant increase in the odds of smokers receiving both cessation advice and a prescription for a smoking cessation medication with increasing deprivation quintile. Patients in the most deprived Townsend quintile were 28% more likely to receive advice compared to those least deprived group (OR 1.28; 95% CI 1.19-1.37), and 16% more likely to receive a prescription (OR 1.16; 95% CI 1.05-1.28).

Table 2 shows the proportion of smokers by Mosaic group who received advice to quit or a cessation medication. There is no natural ranking for Mosaic groups and so odds ratios for both outcomes are presented relative to group A (successful professionals living in desirable areas).

**Table 2:** Odds ratios for the association between Mosaic group and receiving smoking cessation interventions (UK, 2008–10)

**Mosaic group**	**n**	**Advice**			**Prescription**		
		**% received**	OR* (95% CI)	**LRT P-value**	**% received**	OR** (95% CI)	**LRT P-value**
A (Successful professionals living in desirable areas)	21,348	50.1	1.00	< 0.001	13.0	1.00	< 0.001
B (Young families living in new housing estates)	43,373	50.2	1.10 (1.00-1.22)		17.4	1.32 (1.18-1.47)	
C (Older families living in suburbs)	51,081	52.4	1.09 (1.00-1.19)		14.3	1.11 (1.00-1.22)	
D (Close-knit inner city and manufacturing town communities)	66,338	53.0	1.16 (1.05-1.28)		16.9	1.29 (1.14-1.47)	
E (Young, educated and single individuals living in areas of transient populations)	13,352	50.5	1.13 (0.95-1.35)		14.0	1.01 (0.87-1.18)	
F (People with uncertain employment living in social housing in deprived areas)	22,286	57.2	1.35 (1.20-1.52)		17.3	1.28 (1.12-1.47)	
G (Low-income families living in estate based social housing)	37,455	56.2	1.31 (1.17-1.47)		19.5	1.50 (1.31-1.73)	
H (Upwardly mobile families living in former social housing)	65,644	55.1	1.25 (1.13-1.37)		18.3	1.41 (1.26-1.58)	
I (Older people with high care needs living in social housing)	11,889	59.6	1.22 (1.09-1.36)		17.5	1.43 (1.26-1.63)	
J (Independent older people with relatively active lifestyles)	24,355	52.9	1.03 (0.94-1.14)		15.6	1.24 (1.10-1.40)	
K (People living in rural areas far from urbanisation)	14,415	49.9	0.99 (0.88-1.12)		17.0	1.35 (1.16-1.57)	
Missing	89,402	52.1	1.12 (0.97-1.29)		15.5	1.18 (0.99-1.40)	

*adjusted for age, gender and chronic condition **adjusted for age, gender, chronic condition and advice given.

*OR* = odds ratio; 95% CI = 95% confidence interval.

Smokers in Mosaic group A (successful professionals living in desirable areas) had the lowest unadjusted prevalence of receiving a prescription for a smoking cessation medication (13.0%), and the second lowest prevalence of receiving advice to quit (50.1%). Relative to this baseline group, those in Mosaic group F (people with uncertain employment living in social housing in deprived areas) were the most likely to receive advice (OR 1.35; 95% CI 1.20-1.52) and this group also were one of the groups more likely to receive a prescription for a smoking cessation medication. Those most likely to receive a prescription were smokers in group G (low-income families living in estate based social housing), who were 50% more likely to receive a prescription than those in group A (OR 1.50; 95% CI 1.31-1.73).

Table 3 shows the proportion of smokers by Mosaic type who received advice to quit or a smoking cessation medication. Given the large number of categories of Mosaic type, and the impossibility of ranking them in order of deprivation, odds ratios have been calculated using the type with the lowest crude prevalence of the outcome as the baseline category. For brevity we have presented only the 10% of Mosaic types with the lowest and highest adjusted odds ratios; results for all 61 Mosaic types can be viewed in the accompanying Additional file 1: Table S1.

**Table 3:** Odds ratios for the association between Mosaic type and receiving smoking cessation interventions (UK, 2008–10)

	**Advice**				**Prescribing**		
**Mosaic Types ordered from lowest to highest OR**	**N (% received)**	OR* (95% CI)	**LRT P-value**	**Mosaic Types ordered from lowest to highest OR**	**N (% received)**	OR** (95% CI)	**LRT P-value**
E34 (Halls of residence and other buildings occupied mostly by students)	393 (41.5)	1.00	< 0.001	A01 (Financially successful people living in smart flats in cosmopolitan inner city locations)	375 (6.9)	-	< 0.001
K61 (Low income farmers struggling on thin soils in isolated upland locations)	725 (46.6)	1.15 (0.86-1.53)		E29 (Economically successful singles living in privately rented inner city flats)	1,583 (9.9)	1.56 (1.13-2.17)	
A03 (Successful managers living in large housing in outer suburbia)	2,651 (48.0)	1.17 (0.86-1.59)		A03 (Successful managers living in large housing in outer suburbia)	2,651 (9.5)	1.75 (0.96-3.21)	
A04 (Financially secure couples, many close to retirement, living in desirable suburbs)	2,306 (49.8)	1.20 (0.91-1.58)		C20 (Successful members of the Asian community living in suburbs)	3,585 (10.7)	1.84 (1.08-3.14)	
K57 (Communities of retired people and second home owners in areas of high environmental quality)	784 (49.1)	1.22 (0.91-1.65)		A02 (Highly educated senior professionals mainly working in media, politics and law)	1,537 (11.2)	2.02 (1.25-3.26)	
A02 (Highly educated senior professionals mainly working in media, politics and law)	1,537 (47.8)	1.22 (0.86-1.74)		F36 (High density social housing with high levels of diversity, mainly in inner London)	2,983 (12.5)	2.09 (1.19-3.66)	
D26 (Communities of low paid factory workers, many of South Asian descent)	1,250 (55.4)	1.75 (1.36-2.25)		G43 (Elderly, many in poor health due to work in heavy industry, in low rise social housing)	13,334 (19.5)	3.67 (2.27-5.94)	
F36 (High density social housing with high levels of diversity, mainly in inner London)	2,983 (56.9)	1.76 (1.29-2.41)		B08 (Families and singles living in developments built after 2001)	1,399 (19.7)	3.70 (2.27-6.03)	
D27 (Second generation settlers from diverse communities living in multi-cultural inner city terraces)	5,177 (57.3)	1.80 (1.39-2.33)		J56 (Neighbourhoods with retired people and transient singles, working in the health industry)	1,652 (18.6)	3.71 (2.19-6.29)	
F38 (Singles, childless couples and elderly, living in high rise social housing)	1,178 (61.4)	1.86 (1.45-2.39)		G41 (Families, many of which are single parent, living in deprived social housing on the edge of regional areas)	7,515 (20.4)	3.73 (2.31-6.03)	
F40 (Older tenements of small private flats, often occupied by highly disadvantaged individuals)	2,627 (61.4)	2.10 (1.52-2.92)		I50 (Elderly receiving care in homes or sheltered accommodation)	3,551 (18.0)	3.78 (2.32-6.15)	
A01 (Financially successful people living in smart flats in cosmopolitan inner city locations)	375 (69.6)	3.56 (1.28-9.12)		K57 (Communities of retired people and second home owners in areas of high environmental quality)	784 (21.1)	4.37 (2.55-7.49)	

*adjusted for age, gender and chronic condition **adjusted for age, gender, chronic condition and advice given.

*OR* = odds ratio; 95% CI = 95% confidence interval.

Smokers in Mosaic type E34 (halls of residence and other buildings occupied mostly by students) had the lowest unadjusted prevalence of receiving cessation advice (41.5%), whereas smokers in type A01 (financially successful people living in smart flats in cosmopolitan inner city locations) had the highest (69.6%). After adjustment for confounders, the odds of smokers classified as type A01 receiving cessation advice was 3.5 times higher than the odds in group E34 (OR 3.56; 95% CI 1.28-9.12).

Conversely, smokers in Mosaic type A01 were least likely to have received a prescription for a smoking cessation medication, with just 6.9% doing so. Smokers in type K57 (communities of retired people and second home owners in areas of high environmental quality) were most likely to receive a prescription (OR 4.37; 95% CI 2.55-7.49).

Descriptive analysis of the characteristics of each Mosaic type, using the Mosaic Multimedia Guide [23], found that smokers least likely to receive cessation interventions were generally well educated, more often than not to at least degree level, were employed in high income jobs and were financially successful and secure, and tended to be married but with no children. These groups did the majority of their grocery shopping at supermarkets including Sainsbury’s, Waitrose and Marks and Spencer, read broadsheet newspapers and specialist magazines such as The Economist and Newsweek, and had broadband internet access.

Those that were most likely to receive advice tended to be single, lone parents, received more benefits, and watched more television. Smokers most likely to receive a prescription for a smoking cessation medication had a low average annual income, were generally aged over 30 or were pensioners, had no children, and were in receipt of government benefits. They also tended to have little or no access to the internet.

## Discussion and conclusions

This study has highlighted wide variations by socioeconomic group, measured in three different ways, in the odds of smokers receiving cessation advice or a prescription for a cessation medication. Overall, our results tell a consistent story – smokers in the more deprived social groups are more likely to receive advice to quit or a medication prescription. However, the use of Mosaic classification compared to Townsend quintile highlights a greater degree of disparity between population subgroups in the proportion of smokers receiving these interventions, and enables identification of particular types of people who do not fit the general pattern.

Our findings concur with previous research showing that those in more deprived socioeconomic groups are more likely to receive advice to quit smoking from their GP [13,24], perhaps suggesting that primary care health professionals are making a specific effort to target these groups in an attempt to reduce smoking-related inequalities. In addition, more deprived smokers may be more likely to want to receive advice on how to quit [24], and GPs may simply be responding to their patients’ wishes. However, analysis by Mosaic type suggests that some more deprived social groups, such as type K61 (low income farmers struggling on thin soils in isolated upland locations), are amongst the least likely to receive cessation advice. This perhaps supports the persistence of the ‘inverse care law’, whereby good quality healthcare services are least accessible to those who most need them [25], and demonstrates the potential utility of Mosaic to identify deviations from the general underlying pattern.

That more deprived groups are the most likely to receive a prescription for a smoking cessation medication may simply reflect their potential eligibility to receive free National Health Service (NHS) prescriptions (saving, at the time of writing, £7.85 per dispensed item in England). However, in Wales, where prescriptions have been free since April 2007, patterns of prescribing by socioeconomic group appear similar to those reported here for the whole of the UK (though the relatively small number of Welsh practices contributing data to THIN limits the power to detect significant differences). It is possible that those who are not receiving a prescription from their GP are buying NRT elsewhere; NRT is available off-the-shelf in pharmacies and supermarkets, and sales here account for approximately half of all NRT use [26]. Bupropion and varenicline must be prescribed and are not available elsewhere. Though prescribing of bupropion is very low (just 0.7% of smokers were prescribed the drug in the two year study period) and there is limited power to compare prescribing across Mosaic groups and types, analysis of varenicline prescribing patterns suggests that the findings reported here are not only a reflection of less deprived groups buying NRT elsewhere. More affluent Mosaic groups, including group A (Successful professionals living in desirable areas) and E (Young, educated and single individuals living in areas of transient populations) were least likely to be prescribed varenicline, in line with the pattern of prescribing of cessation medications overall.

Frequency of consultation with a GP is known to vary by socioeconomic status; in Great Britain adults who are either employed or currently unemployed consult with a GP approximately four times per year, whereas the economically inactive consult seven times per year [27]. A higher consultation frequency in the most deprived groups may increase the number of opportunities for a GP to deliver a cessation intervention, contributing to the higher rates of intervention reported here. Data on consultation rates in THIN patients were not available to us and thus we were not able to take variations into account in our analyses.

To our knowledge this is the first study to investigate variations in the delivery of smoking cessation interventions using Mosaic as a measure of socioeconomic status, and the first using such a large database of patient records. The large dataset affords the power to compare the delivery of cessation interventions in multiple subgroups, though in the case of Mosaic it is not immediately obvious which category to use as the baseline for comparisons. Our use of the group and type with the lowest prevalence of receiving advice and prescribing will serve to maximise the difference in odds ratios between categories, though we feel this is appropriate to highlight the extremes of intervention prevalence identified using Mosaic. Given that the prevalence of cessation intervention is relatively high there may be some discrepancy between our odds ratios and the relative risk. However, this is unlikely to materially change the conclusions of our study [28]. Confidence in Mosaic is limited by the lack of available information about how the classification system is derived, and the large number of Mosaic types means the indicator may be quite cumbersome to use as a measure of socioeconomic status in statistical models or in public health planning. Care must also be taken to avoid the ecological fallacy; Mosaic is an area-level measure, derived at the level of postcode areas representing approximately 15 households, and thus the characteristics of a Mosaic group or type will not apply to all people in that group.

That smokers in more deprived groups are more likely to receive a cessation intervention may help to reduce the smoking-related socioeconomic inequalities in health which result from the higher smoking prevalence in these groups. However, overall just 53.1% of smokers were advised to quit and only 16.5% were prescribed a cessation medication during the two year study period; these proportions highlight room for improvement across all social groups to ensure all smokers receive these effective interventions. The profiles of each Mosaic type provided by Experian suggest ways in which specific groups could be targeted to increase the delivery and uptake of effective interventions. The generally-successful groups who were least likely to receive smoking cessation advice or a prescription for a cessation medication could perhaps best be targeted through media such as the internet or in broadsheet newspapers and specialist magazines; television adverts may not reach these groups as they are generally light viewers. Workplace-based interventions may also be an effective way to reach these groups [29], who are generally all in employment. Further work is warranted to understand the barriers preventing the most financially and socially successful groups accessing the cessation interventions available through primary care, or the GP-level factors limiting the delivery of interventions to smokers in these groups. It is encouraging that the higher prevalence of cessation intervention in the more deprived social groups is unlikely to contribute to a widening of health inequalities. The use of Mosaic as illustrated here demonstrates the potential to market cessation interventions appropriately in different types of smokers in order to increase the overall rate of intervention as well as that in specific groups.

## Competing interests

The authors have no competing interests to declare. Experian, the developers of the Mosaic classification, played no part in the design and implementation of the study, nor the interpretation of results and preparation of the manuscript for publication.

## Authors’ contributions

LS designed the study and prepared the data for analysis. LD carried out the statistical modelling and prepared the first draft of the manuscript. Both authors contributed to the final manuscript and have approved its publication.

## Pre-publication history

The pre-publication history for this paper can be accessed here:

http://www.biomedcentral.com/1471-2458/13/546/prepub

## Supplementary Material

Additional file 1: Table S1 Odds ratios for the association between Mosaic type and receiving smoking cessation interventions for all 61 Mosaic types (UK, 2008-10). Click here for file
