# Supplementary material for: Socioeconomic variations in access to smoking cessation interventions in UK primary care: insights using the Mosaic classification in a large dataset of primary care records
Source: BMC Public Health. 2013 Jun 5;13:546. doi: 10.1186/1471-2458-13-546 (PMC3710237; doi:10.1186/1471-2458-13-546)
Supplement: Additional file 1: Table S1 — Odds ratios for the association between Mosaic type and receiving smoking cessation interventions for all 61 Mosaic types (UK, 2008-10). [file 1471-2458-13-546-S1.docx]

Additional file 1: Odds ratios for the association between Mosaic type and receiving smoking cessation interventions for all 61 Mosaic types (UK, 2008-10)

| **Advice** | | | | **Prescribing** | | | |
| --- | --- | --- | --- | --- | --- | --- | --- |
| **Mosaic Type** | **N**  **(% received)** | **OR***  **(95% CI)** | **LRT P-value** | **Mosaic Type** | **N**  **(% received)** | **OR****  **(95% CI)** | **LRT P-value** |
| E34 (Halls of residence and other building occupied mostly by students) | 393  (41.5) | 1.00 | <0.001 | A01 (Financially successful people living in smart flats in cosmopolitan inner city locations) | 375  (6.9) | - | <0.001 |
| K61 (Low income farmers struggling on thin soils in isolated upland locations) | 725  (46.6) | 1.15  (0.86-1.53) |  | E29 (Economically successful singles living in privately rented inner city flats) | 1,583  (9.9) | 1.56  (1.13-2.17) |  |
| A03 (Successful managers living in large housing in outer suburbia) | 2,651  (48.0) | 1.17  (0.86-1.59) |  | A03 (Successful managers living in large housing in outer suburbia) | 2,651  (9.5) | 1.75  (0.96-3.21) |  |
| A04 (Financially secure couples, many close to retirement, living in desirable suburbs) | 2,306  (49.8) | 1.20  (0.91-1.58) |  | C20 (Successful members of the Asian community living in suburbs) | 3,585  (10.7) | 1.84  (1.08-3.14) |  |
| K57 (Communities of retired people and second home owners in areas of high environmental quality) | 784  (49.1) | 1.22  (0.91-1.65) |  | A02 (Highly educated senior professionals mainly working in media, politics and law) | 1,537  (11.2) | 2.02  (1.25-3.26) |  |
| A02 (Highly educated senior professionals mainly working in media, politics and law) | 1,537  (47.8) | 1.22  (0.86-1.74) |  | F36 (High density social housing with high levels of diversity, mainly in inner London) | 2,983  (12.5) | 2.09  (1.19-3.66) |  |
| K58 (Financially well-off commuters and retired people living in attractive village locations) | 4,549  (49.3) | 1.24  (0.97-1.58) |  | A04 (Financially secure couples, many close to retirement, living in desirable suburbs) | 2,306  (10.9) | 2.09  (1.27-3.44) |  |
| J51 (Very elderly people, many financially secure, living in privately owned retirement flats) | 1,296  (55.8) | 1.24  (0.97-1.58) |  | D27 (Second generation settlers from diverse communities living in multi-cultural inner city terraces) | 5,177  (12.5) | 2.09  (1.16-3.77) |  |
| J56 (Neighbourhoods with retired people and transient singles, working in the health industry) | 1,652  (49.8) | 1.25  (0.94-1.65) |  | D26 (Communities of low paid factory workers, many of South Asian descent) | 1,250  (12.6) | 2.17  (1.30-3.62) |  |
| J53 (Financially secure and physically active elderly, many retired to semi-rural locations) | 3,645  (52.4) | 1.26  (1.00-1.58) |  | E30 (Young professional families living in gentrifies terraces in pre-1914 suburbs) | 2,560  (12.8) | 2.17  (1.49-3.17) |  |
| A05 (Senior professionals and managers living in suburbs of major regional centres) | 3,775  (50.7) | 1.26  (0.99-1.60) |  | E34 (Halls of residence and other building occupied mostly by students) | 393  (11.2) | 2.25  (1.26-4.02) |  |
| A07 (Well paid executives living in individually designed homes in rural locations) | 4,833  (49.7) | 1.27  (1.01-1.61) |  | J52 (Older people, singles and childless couples in developments of private flats) | 3,294  (12.6) | 2.28  (1.39-3.76) |  |
| J54 (Older independent couples on limited incomes, living in bungalows by the sea) | 3,699  (54.5) | 1.28  (1.02-1.62) |  | E28 (Neighbourhoods with transient singles living in multiply occupied large old houses) | 2,085  (13.6) | 2.31  (1.48-3.60) |  |
| E33 (Older neighbourhoods with increasing numbers of short term student renters) | 1,182  (47.6) | 1.29  (1.04-1.60) |  | E33 (Older neighbourhoods with increasing numbers of short term student renters) | 1,182  (12.9) | 2.40  (1.41-4.09) |  |
| C15 (Senior white collar workers, many on the verge of a financially secure retirement) | 10,084  (50.2) | 1.30  (1.02-1.66) |  | C19 (Families, singles and childless couples living in attractive older suburbs) | 8,075  (13.5) | 2.45  (1.48-4.05) |  |
| J52 (Older people, singles and childless couples in developments of private flats) | 3,294  (50.7) | 1.30  (1.02-1.66) |  | A05 (Senior professionals and managers living in suburbs of major regional centres) | 3,775  (13.2) | 2.50  (1.53-4.07) |  |
| K59 (Country people living in agriculturally active villages, mostly in lowland locations) | 5,116  (50.2) | 1.31  (1.05-1.65) |  | C15 (Senior white collar workers, many on the verge of a financially secure retirement) | 10,084  (13.5) | 2.54  (1.57-4.11) |  |
| E31 (Well educated singles and childless couples living in inner areas of provincial cities) | 2,402  (49.0) | 1.32  (1.04-1.67) |  | A06 (Successful couples working in areas of growing high tech employment) | 5,871  (14.4) | 2.68  (1.65-4.36) |  |
| D25 (Centres of small market towns containing many hostels and refuges) | 4,973  (50.0) | 1.33  (1.06-1.68) |  | C16 (Self-reliant couples approaching retirement, living in low density private estates) | 7,633  (14.0) | 2.69  (1.65-4.37) |  |
| C16 (Self-reliant couples approaching retirement, living in low density private estates) | 7,633  (53.9) | 1.34  (1.06-1.71) |  | F38 (Singles, childless couples and elderly, living in high rise social housing) | 1,178  (15.7) | 2.70  (1.64-4.45) |  |
| C19 (Families, singles and childless couples living in attractive older suburbs) | 8,075  (50.1) | 1.35  (1.05-1.73) |  | F35 (Young people renting social housing often in disadvantages inner city locations) | 1,646  (15.4) | 2.71  (1.58-4.65) |  |
| K60 (Smallholders and self-employed farmers, living beyond the reach of urban communities) | 3,241  (51.2) | 1.36  (1.07-1.74) |  | J54 (Older independent couples on limited incomes, living in bungalows by the sea) | 3,699  (14.0) | 2.76  (1.65-4.61) |  |
| B10 (Financially well-off families living in modern private estates) | 3,684  (48.5) | 1.37  (1.08-1.72) |  | A07 (Well paid executives living in individually designed homes in rural locations) | 4,833  (14.9) | 2.77  (1.68-4.55) |  |
| A06 (Successful couples working in areas of growing high tech employment) | 5,871  (50.5) | 1.37  (1.08-1.74) |  | Missing | 89,402  (15.5) | 2.84  (1.75-4.61) |  |
| B12 (Middle income families with children living in modern private estates) | 14,667  (50.2) | 1.38  (1.09-1.73) |  | K58 (Financially well-off commuters and retired people living in attractive village locations) | 4,549  (15.0) | 2.85  (1.73-4.69) |  |
| B11 (Dual income families of intermediate incomes living in modern estates) | 8,562  (49.7) | 1.39  (1.11-1.75) |  | E31 (Well educated singles and childless couples living in inner areas of provincial cities) | 2,402  (15.9) | 2.85  (1.63-5.01) |  |
| C17 (Small business proprietors living in low density estates in smaller communities) | 9,790  (52.2) | 1.40  (1.11-1.75) |  | C18 (Inter-war suburbs many with less cohesion than originally had) | 11,914  (15.5) | 2.87  (1.76-4.68) |  |
| D21 (Mixed communities of urban residents living in well-built early 20^th^ century housing) | 10,138  (51.3) | 1.42  (1.13-1.78) |  | E32 (Singles and childless couples living in small units in new private estates) | 3,147  (16.7) | 2.92  (1.78-4.79) |  |
| J55 (Older people living in familiar small market towns) | 10,769  (53.3) | 1.42  (1.13-1.79) |  | K61 (Low income farmers struggling on thin soils in isolated upland locations) | 725  (15.5) | 2.94  (1.66-5.22) |  |
| Missing | 89,402  (52.1) | 1.45  (1.14-1.84) |  | D22 (Financially comfortable manual workers living in inexpensive but spacious private houses) | 13,179  (15.8) | 2.95  (1.82-4.77) |  |
| D22 (Financially comfortable manual workers living in inexpensive but spacious private houses) | 13,179  (53.7) | 1.47  (1.17-1.84) |  | J53 (Financially secure and physically active elderly, many retired to semi-rural locations) | 3,645  (15.0) | 2.95  (1.79-4.89) |  |
| E30 (Young professional families living in gentrifies terraces in pre-1914 suburbs) | 2,560  (50.6) | 1.49  (0.96-2.32) |  | B12 (Middle income families with children living in modern private estates) | 14,667  (16.2) | 2.96  (1.83-4.78) |  |
| B13 (First generation owner occupiers, many with large consumer debts) | 9,807  (50.8) | 1.49  (1.17-1.89) |  | C17 (Small business proprietors living in low density estates in smaller communities) | 9,790  (16.1) | 3.01  (1.86-4.87) |  |
| H47 (Social housing, typically in ‘new towns’ with good job opportunities for the poorly qualified) | 16,287  (51.9) | 1.50  (1.20-1.89) |  | D21 (Mixed communities of urban residents living in well-built early 20^th^ century housing) | 10,138  (16.6) | 3.02  (1.87-4.90) |  |
| D24 (Low income families living in cramped Victorian terraces in inner city locations) | 12,807  (52.3) | 1.51  (1.20-1.90) |  | H46 (Residents in 1930s and 1950s council estates, typically in London, now mostly owner occupiers) | 19,588  (16.7) | 3.04  (1.85-4.99) |  |
| D23 (Owners of affordable terraces built to house 19^th^ century heavy industrial workers) | 18,814  (53.2) | 1.53  (1.22-1.91) |  | F40 (Older tenements of small private flats, often occupies by highly disadvantaged individuals) | 2,627  (17.3) | 3.05  (1.87-4.98) |  |
| B09 (Well qualified couples, many starting a family, living on a new private estate) | 4,918  (50.9) | 1.53  (1.22-1.92) |  | B14 (Military personal living in purpose built accommodation) | 336  (17.9) | 3.10  (1.78-5.41) |  |
| E28 (Neighbourhoods with transient singles living in multiply occupied large old houses) | 2,085  (52.4) | 1.54  (1.06-2.23) |  | J55 (Older people living in familiar small market towns) | 10,769  (16.6) | 3.12  (1.93-5.05) |  |
| C18 (Inter-war suburbs many with less cohesion than originally had) | 11,914  (54.3) | 1.54  (1.22-1.93) |  | I48 (Elderly living in small council and housing association flats) | 2,170  (15.8) | 3.13  (1.90-5.14) |  |
| E32 (Singles and childless couples living in small units in new private estates) | 3,147  (51.8) | 1.54  (1.22-1.94) |  | B10 (Financially well-off families living in modern private estates) | 3,684  (17.1) | 3.14  (1.92-5.12) |  |
| I50 (Elderly receiving care in homes or sheltered accommodation) | 3,551  (63.4) | 1.56  (1.25-1.95) |  | B11 (Dual income families of intermediate incomes living in modern estates) | 8,562  (17.3) | 3.19  (1.97-5.17) |  |
| B08 (Families and singles living in developments built after 2001) | 1,399  (50.6) | 1.56  (1.24-1.97) |  | D24 (Low income families living in cramped Victorian terraces in inner city locations) | 12,807  (18.0) | 3.30  (2.04-5.34) |  |
| I49 (Low income older couples renting low rise social housing in industrial regions) | 6,168  (56.1) | 1.57  (1.25-1.97) |  | B13 (First generation owner occupiers, many with large consumer debts) | 9,807(18.3) | 3.31  (2.05-5.35) |  |
| F35 (Young people renting social housing often in disadvantages inner city locations) | 1,646  (53.3) | 1.58  (1.25-1.99) |  | B09 (Well qualified couples, many starting a family, living on a new private estate) | 4,918  (18.8) | 3.35  (2.04-5.50) |  |
| H46 (Residents in 1930s and 1950s council estates, typically in London, now mostly owner occupiers) | 19,588  (54.8) | 1.60  (1.27-2.01) |  | K59 (Country people living in agriculturally active villages, mostly in lowland locations) | 5,116  (17.7) | 3.37  (2.07-5.48) |  |
| H45 (Older couples living, mostly in small towns, in houses formerly rented from the council) | 11,552  (56.9) | 1.62  (1.31-2.01) |  | I49 (Low income older couples renting low rise social housing in industrial regions) | 6,168  (17.9) | 3.37  (2.08-5.47) |  |
| I48 (Elderly living in small council and housing association flats) | 2,170  (63.3) | 1.64  (1.30-2.06) |  | F39 (Older people living in apartments in high density social housing) | 5,869  (18.3) | 3.38  (2.07-5.52) |  |
| C20 (Successful members of the Asian community living in suburbs) | 3,585  (54.1) | 1.64  (1.26-2.13) |  | D25 (Centres of small market towns containing many hostels and refuges) | 4,973  (18.0) | 3.39  (2.09-5.52) |  |
| F37 (Young families living in upper floors of social housing) | 7,983  (55.1) | 1.69  (1.35-2.11) |  | D23 (Owners of affordable terraces built to house 19^th^ century heavy industrial workers) | 18,814  (18.4) | 3.42  (2.12-5.52) |  |
| G42 (Families with school-age children, living in large social housing estates on the outskirts of provincial cities) | 16,606  (55.7) | 1.69  (1.35-2.11) |  | F37 (Young families living in upper floors of social housing) | 7,983  (18.9) | 3.42  (2.12-5.52) |  |
| G43 (Elderly, many in poor health due to work in heavy industry, in low rise social housing) | 13,334  (57.7) | 1.70  (1.35-2.13) |  | K60 (Smallholders and self-employed farmers, living beyond the reach of urban communities) | 3,241  (17.9) | 3.44  (2.09-5.64) |  |
| B14 (Military personal living in purpose built accommodation) | 336  (53.9) | 1.73  (1.23-2.43) |  | H45 (Older couples living, mostly in small towns, in houses formerly rented from the council) | 11,552  (18.2) | 3.45  (2.13-5.59) |  |
| G41 (Families, many of which are single parent, living in deprived social housing on the edge of regional areas) | 7,515  (54.8) | 1.73  (1.39-2.15) |  | G42 (Families with school-age children, living in large social housing estates on the outskirts of provincial cities) | 16,606  (19.1) | 3.52  (2.17-5.71) |  |
| H44 (Manual workers, many close to retirement, living in low rise houses of ex-manufacturing towns) | 18,217  (57.2) | 1.73  (1.39-2.16) |  | J51 (Very elderly people, many financially secure, living in privately owned retirement flats) | 1,296  (16.7) | 3.53  (2.14-5.82) |  |
| F39 (Older people living in apartments in high density social housing) | 5,869  (58.8) | 1.74  (1.37-2.20) |  | H44 (Manual workers, many close to retirement, living in low rise houses of ex-manufacturing towns) | 18,217  (19.1) | 3.54  (2.18-5.73) |  |
| E29 (Economically successful singles living in privately rented inner city flats) | 1,583  (52.1) | 1.74  (0.81-3.74) |  | H47 (Social housing, typically in ‘new towns’ with good job opportunities for the poorly qualified) | 16,287  (19.3) | 3.61  (2.24-5.80) |  |
| D26 (Communities of low paid factory workers, many of South Asian descent) | 1,250  (55.4) | 1.75  (1.36-2.25) |  | G43 (Elderly, many in poor health due to work in heavy industry, in low rise social housing) | 13,334  (19.5) | 3.67  (2.27-5.94) |  |
| F36 (High density social housing with high levels of diversity, mainly in inner London) | 2,983  (56.9) | 1.76  (1.29-2.41) |  | B08 (Families and singles living in developments built after 2001) | 1,399  (19.7) | 3.70  (2.27-6.03) |  |
| D27 (Second generation settlers from diverse communities living in multi-cultural inner city terraces) | 5,177  (57.3) | 1.80  (1.39-2.33) |  | J56 (Neighbourhoods with retired people and transient singles, working in the health industry) | 1,652  (18.6) | 3.71  (2.19-6.29) |  |
| F38 (Singles, childless couples and elderly, living in high rise social housing) | 1,178  (60.5) | 1.86  (1.45-2.39) |  | G41 (Families, many of which are single parent, living in deprived social housing on the edge of regional areas) | 7,515  (20.4) | 3.73  (2.31-6.03) |  |
| F40 (Older tenements of small private flats, often occupies by highly disadvantaged individuals) | 2,627  (61.4) | 2.10  (1.52-2.92) |  | I50 (Elderly receiving care in homes or sheltered accommodation) | 3,551  (18.0) | 3.78  (2.32-6.15) |  |
| A01 (Financially successful people living in smart flats in cosmopolitan inner city locations) | 375  (69.6) | 3.56  (1.28-9.92) |  | K57 (Communities of retired people and second home owners in areas of high environmental quality) | 784  (21.1) | 4.37  (2.55-7.49) |  |

*adjusted for age, gender and chronic condition **adjusted for age, gender, chronic condition and advice given

OR = odds ratio; 95% CI = 95% confidence interval
